# Conducting mixed-methods research with Ebola survivors in a complex setting in Sierra Leone

**DOI:** 10.1186/s12889-020-09469-9

**Published:** 2020-09-03

**Authors:** Soumya Alva, Nicole Davis, Laurentiu Stan, Isotta Pivato, Jeffrey Sanderson

**Affiliations:** 1 John Snow, Inc., Arlington, Virginia USA; 2 RTI International, Durham, North Carolina USA; 3 Relief International, Istanbul, Turkey

**Keywords:** Mixed-methods, Sierra Leone, Ebola, Mental health

## Abstract

**Background:**

In late 2015, the Sierra Leone government established the Comprehensive Program for Ebola Survivors (CPES) to improve the well-being of 3466 registered Ebola virus disease (EVD) survivors. This case analysis outlines the challenges of conducting research studies on the health situation of these EVD survivors in a complicated, post-Ebola context. It outlines strategies to address these challenges without compromising research quality. The mixed-methods study sought to determine EVD survivors’ access to health services offered through CPES, their health and disability status, and psychosocial and mental health issues faced. Qualitative data from survivors and stakeholders at multiple levels complemented and contextualized the survey results to help understand the unique health and associated socioeconomic challenges that EVD survivors face, which could be applied to other crisis settings. Study findings indicated that CPES had lasting impacts on Sierra Leone’s health system, enabling it to respond to EVD survivors, who increasingly accessed health services and showed lower levels of disability after receiving care.

**Discussion:**

Understanding the health service needs of this specialized population in a country with an overloaded health system after the Ebola epidemic makes this research study important and timely. The study faced several challenges, including working in a low-resource and low-capacity setting marked by constantly changing priorities and activities of CPES donors and implementers. Further, the study aimed to measure sensitive topics, such as mental health and disability, with standardized tools that required careful contextualization for accurate reporting of findings. Strategies to overcome these challenges included utilizing a mixed-methods approach to contextualize and validate survey results. The study also enabled capacity building of local research teams to ensure that they could follow lines of inquiry and navigate the complex post-Ebola context.

**Conclusions:**

Flexibility is paramount when conducting high-quality research for representative and useful results. Timely research and ongoing sharing of the findings with stakeholders is critical to ensure that they benefit study subjects. Furthermore, in such settings, there is a need to balance engagement of stakeholders with maintaining independence and impartiality in the research design and subsequent data produced.

## Background

### Humanitarian context

The spread of the Ebola epidemic in Sierra Leone and the neighboring countries of Liberia and Guinea resulted in more than 11,000 deaths across the region by March 2016 [1]. In Sierra Leone alone, there were more than 14,000 cases and 3956 deaths [1]. The epidemic led to the breakdown of the health system, with the death of health staff and a decline in the population’s trust in available health services [2, 3]. Ministry of Health and Sanitation (MOHS) health facilities were modified throughout the country in order to treat and refer Ebola Virus Disease (EVD) survivors after their discharge from Ebola Treatment Units. Many non-governmental organizations supported temporary specialty clinical services at care centers to serve this population. The response to Ebola focused mainly on disease treatment, improving infection prevention and control practices, and reconstruction of the health system.

Studies show that EVD survivors face lifelong health complications such as mental health issues, joint pain, vision problems, among other health issues [4–9]. The chronic conditions that survivors face require ongoing care at local health facilities or secondary/tertiary care (e.g. mental healthcare) that are accessible only in the country’s main towns. Recent studies have described variability in health-seeking behavior of survivors for post EVD care as well as experiences of health workers who faced limited availability of medicines and challenges with referrals to specialists in order to provide this care [10, 11]. Additionally, Ebola-related deaths of household heads and the poor health of survivors led to loss of livelihood among households in the affected regions in addition to orphaned children [9].

In late 2015, the Sierra Leone government-mandated national program, Comprehensive Program for Ebola Survivors (CPES), received UK and US government and donor support to improve the well-being of 3466 registered EVD survivors in 12 districts through the provision of basic and specialized healthcare, allowing them to access public-sector health services without cost^1^ [12]. Specialized care provided to survivors covered ophthalmology, neurology, mental health, reproductive and child health, as well as counseling, semen testing, and treatment for other EVD sequelae. They were also beneficiaries of the existing MOHS-led Free Healthcare Initiative that was designed for pregnant women, lactating mothers and children under-five.

**Fig. 1:**
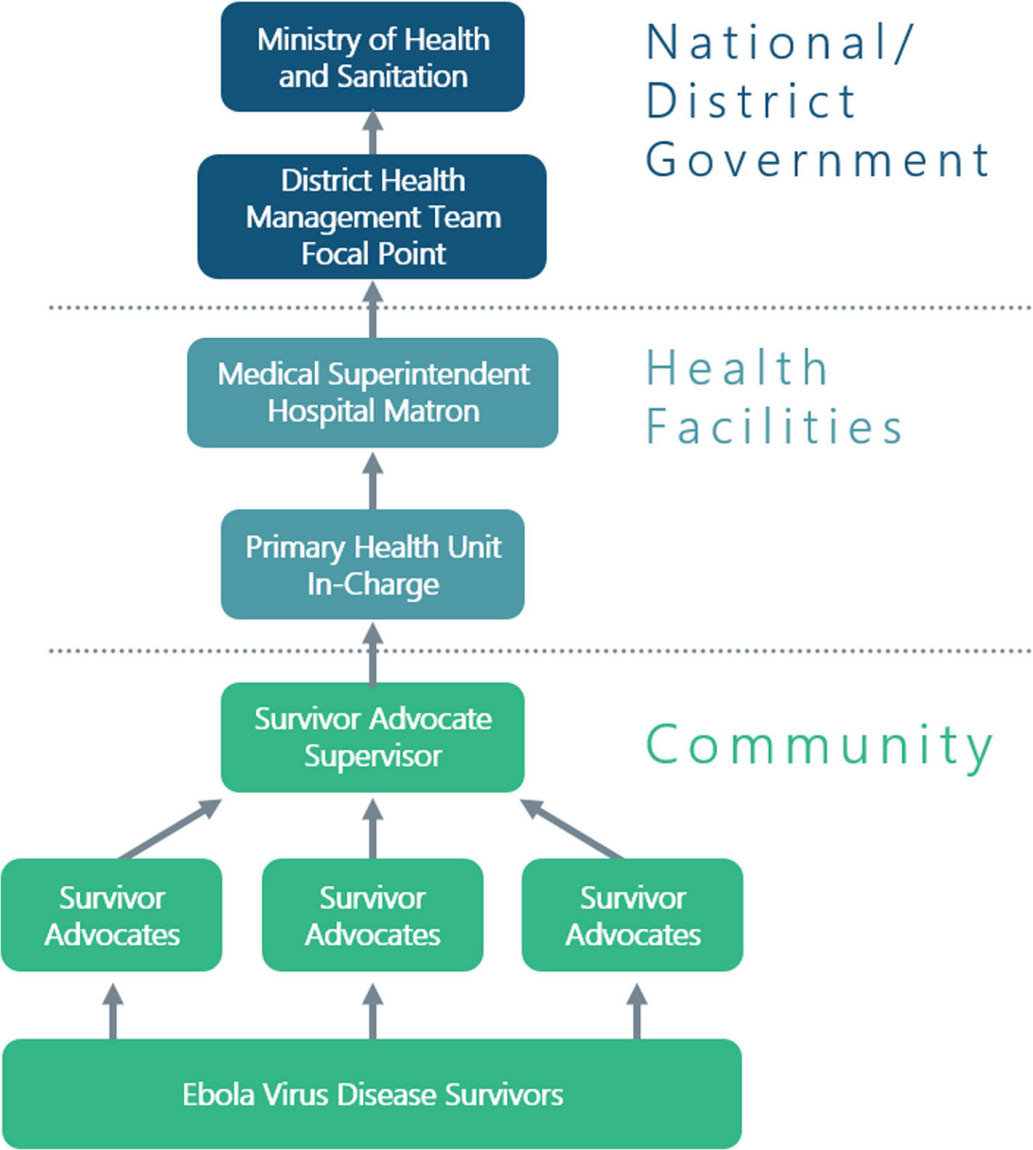
CPES Implementation Structure. *Source: CPES*

While CPES built the capacity of healthcare workers and supported access to basic health services in facilities at the district and lower levels, specialty services were strengthened at the district and tertiary hospitals. A team of “Survivor Advocates”, who were themselves EVD survivors, provided community-level social support (see Fig. 1).

**Fig. 2:**
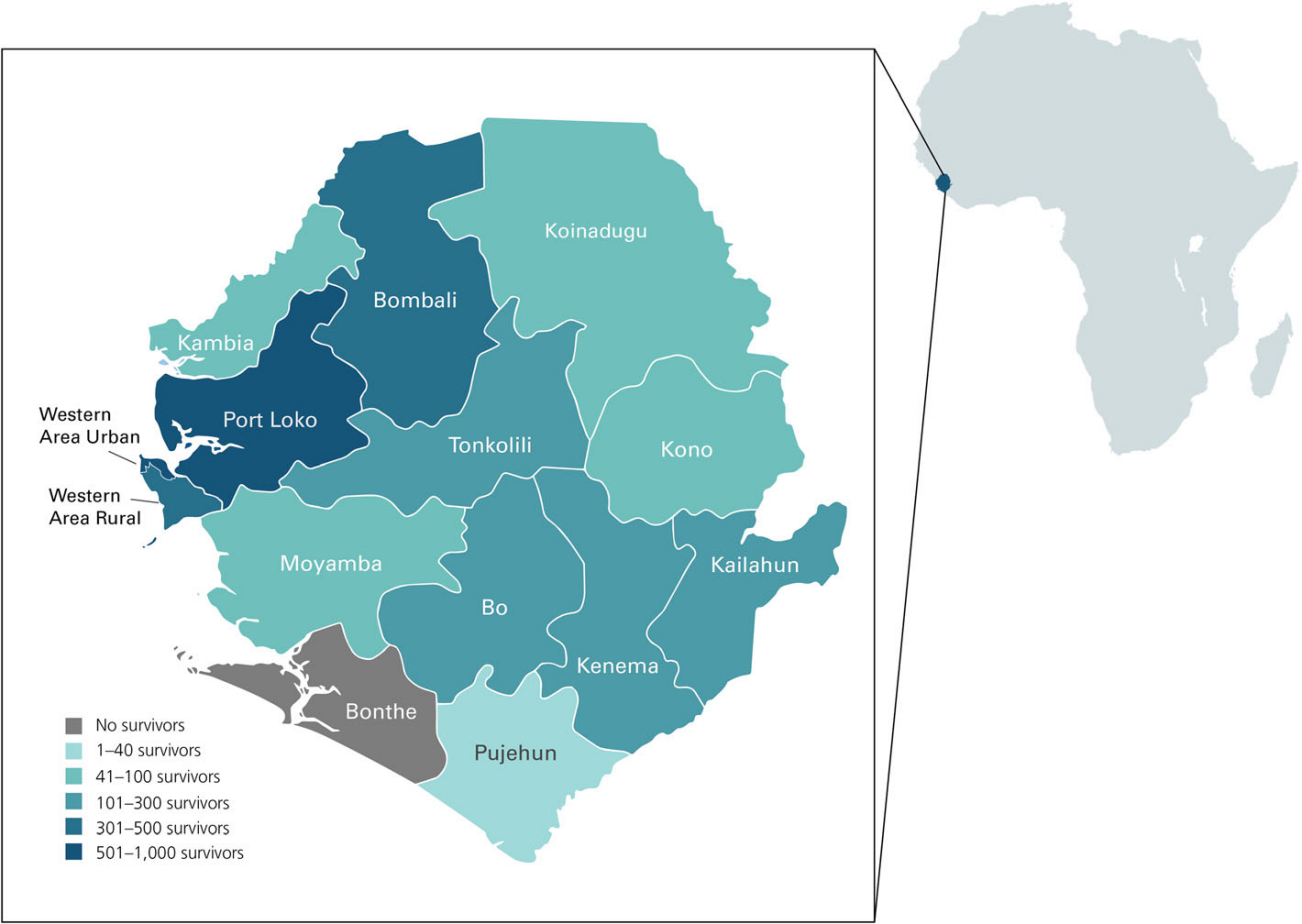
Location of registered EVD survivors in Sierra Leone. *Source: The district-level map of Sierra Leone is based on spatial data from the 2013 District Health Survey* (https://spatialdata.dhsprogram.com/boundaries/#view=map&countryId=SL&surveyId=450&level=2). *The concentration of Ebola survivors in each district is based on data from Sierra Leone’s Ministry of Social Welfare, Gender, and Children’s Affairs Database*

The intended beneficiary population of the program was EVD survivors who, as a group, were represented by the civil society organization Sierra Leone Association of Ebola Survivors (SLAES). SLAES worked with established government networks and implementing partners (IPs) to advocate for the needs of EVD survivors. Figure 2 presents an overview of the location of these survivors in the country based on these data. EVD survivors were spread across the country, but mostly concentrated in three districts - Western Area Urban, Western Area Rural, and Port Loko.

### Research study

A mixed-methods research study [13] was conducted culminating in 2018 after 2 years of CPES program implementation with an endline assessment to understand the role of services provided by the program and the resulting health situation of EVD survivors, as measured by their disability and mental health status. Specific questions for the study included:

- What services did EVD survivors receive through CPES? Were they satisfied with these services?
- What barriers did EVD survivors face in accessing health services?
- What was the nature of stigma EVD survivors faced while accessing health services?
- What was the change in disability status of EVD survivors?
- What mental health and psycho-social conditions were faced by EVD survivors?

To generate nationally representative results, the study used the lot quality assurance sampling (LQAS) methodology and oversampled in the three districts with high numbers of EVD survivors, resulting in a study population of 751 male and female EVD survivors aged 18 and older [14–16]. Key informant interviews were conducted with members of SLAES, district and health facility staff, and survivors to provide additional context to the survey results. Though similar to the baseline, the endline questionnaires included additional questions on the effect of CPES programming on the beneficiaries. Change in survivors’ disability status was assessed based on survivors’ self-reporting using the WHO Disability Assessment Schedule Short version (WHODAS-Short) [17]. The endline also included an additional mental health module using the Patient Health Questionnaire 9 (PHQ-9) and General Anxiety Disorder 7 (GAD-7) to understand the mental health status of survivors, a topic that was not covered at baseline [18, 19].

Research findings showed that in the 15 months between baseline and endline, the proportion of EVD survivors seeking care at a health facility for post-Ebola health issues increased from 89 to 96% (*p* < .001). The proportion of those referred to a higher-level facility to receive appropriate care remained the same at just under one-third of EVD survivors, of which almost all reported that they attended the referral facility.

The program had lasting impacts on the ability of Sierra Leone’s health system to respond to the needs of EVD survivors. Health facility staff indicated that the training and mentorship received over the course of the program made them more capable of dealing with the basic health needs of survivors, particularly when it came to identifying and addressing mental health issues. However, funding and programmatic changes over the life of the project led to a decrease in the availability of medications at the health facilities or peripheral health units (PHUs). This may have influenced EVD survivors’ perceptions of the quality of care they received at PHUs between baseline and endline. Despite lower perceptions of quality of care received, EVD survivors reported lower levels of stigma (12% decrease) during their interactions with health workers between baseline and endline.

Survivors were screened for both anxiety and depression during the endline assessment. Thirty-two percent of survivors exhibited symptoms of mild anxiety and just over 10 % exhibited signs of moderate or severe anxiety. Similarly, 29% of survivors exhibited signs of mild depression and 18% had signs of moderate to severe depression. Further, the proportion of EVD survivors experiencing some level of disability dropped from 41 to 30% (*p* < .001) between baseline and endline.

## Discussion

### Scientific importance of this research

Given this scenario of a population with special health service needs in a country context with an overloaded health system after the Ebola epidemic, this research study on the situation of survivors and the health services that they access and receive is especially important and timely. The study worked with a specific population believed to face mental health challenges and stigma when accessing health services. The study highlights methods by which populations facing challenges in the post-Ebola context can be effectively assessed. The study also adapted generic tools such as the WHODAS and the mental health modules (PHQ-9 and GAD-7) to the country context to examine effects of the epidemic on the disability and mental health status of survivors in the West Africa post-Ebola setting. These tools had to take into account population knowledge, language and literacy levels, as well as cultural understanding to ensure that the respondents interpreted them as intended. Care was also needed to ensure that the study population would be receptive to answering questions of this nature. The study provides an example of how such adaptations may take place in other developing country contexts facing similar concerns.

Within Sierra Leone, research findings were especially useful to the MOHS and other stakeholders, all of whom contributed to the design and implementation of this study through regular discussions within the CPES Program Implementation Unit (PIU) and IPs during project implementation. To ensure timely and widespread sharing of results, study findings were disseminated at various national- and global-level venues. This included sharing results with the CPES program implementation unit and other country stakeholders as well as presenting findings at international conferences to inform global audiences.

With recurring Ebola epidemics within the African continent and the evolving knowledge on EVD survivors, findings from this research study are globally relevant. Although EVD survivors form a small percentage of the country population, countries experiencing Ebola outbreaks need to be prepared for how they will address the short- and long-term health and livelihood needs of this population, particularly within the context of weak health systems that will only be further strained and burdened by Ebola.

### Challenges to research

#### Inconsistent program implementation

Conducting this research study in the Sierra Leone post-Ebola context was not without its challenges. Despite being a national, MOHS-led program, CPES was predominantly donor-funded and implemented by several IPs working in one or more districts. Although they were expected to follow a standard implementation process in each district, small differences existed across the districts based on the situation on the ground, which needed to be taken into account when contextualizing the results of the research. For example, in some districts, one of the IPs paid for drugs obtained by patients from private pharmacies when they were not available at the PHUs. This was not the case in other districts, resulting in differences among survivors’ expectations and perceptions of services provided by CPES. Furthermore, the program design was not inherently sustainable because of varied funding over the life of the project, affecting survivor services, and as a result, survivor perceptions and experience of service availability. These factors influenced the research team’s analysis and triangulation of data collected for the study.

#### Logistics

As with most studies in low-resource settings, logistics to conduct the study posed different challenges at both baseline and endline. At baseline, IPs were already engaging with survivors, which in some ways made it easier for the research team to contact and transport sampled survivors to be interviewed. However, ensuring consistency of sampling procedures required additional coordination and communication because they were conducted by different IPs in different districts across the country. Conversely, at the time of endline, programmatic changes required the research team to work with SLAES to mobilize the randomly selected survivors to participate in the study. Because of the potential for SLAES as an advocacy organization to introduce bias in survivor responses, the research team had to ensure that although SLAES played a prominent role in mobilizing respondents, it was not involved in the actual data collection process.

#### Limited scope of study

The post-Ebola context of Sierra Leone left EVD survivors recovering from often dire consequences of the outbreak. Both CPES and this research study focused on EVD survivors’ health, leaving out their livelihood needs. The impact of this was twofold; it limited the breadth of information collected about and from survivors, which could fully contextualize the results of the study, and it limited the impact of our research on EVD survivors because of its focus on a subset of their post-Ebola needs.

#### Contextualization of assessment tools

A final challenge to the research was the use of mental health and disability assessment tools (WHODAS-Short, PHQ-9, and GAD-7). Assessment and diagnosis of mental health- and disability-related issues varies from country to country, making it difficult to draw definitive conclusions. Globally validated tools that were used posed sensitive questions, particularly to EVD survivor population who were stressed and impacted by many elements of the context at the time of the study. Thus, effective implementation of the tools required careful translation into regional languages of Krio, Mende, and Temne to capture accurate measures for disability, anxiety, and depression among EVD survivors and ensure that EVD survivors were comfortable fully engaging in the survey. Many medical terms or concepts used in the study tools were difficult to translate into these languages requiring extensive rounds of verbal translation and back translation among the data collection teams. This process allowed for discussion among the team, which helped to ensure clarity of concepts as well as consistency on how each question was asked across the three languages.

### Research strategies

#### Sampling strategy

There was a strong desire for results of the survivor survey to be nationally representative; however, traditional sampling methodology would require the majority of EVD survivors in each district to be interviewed. Moreover, as Fig. 2 shows, the distribution of survivors was very uneven in districts across the country. As a result, the research team chose to utilize the LQAS methodology to determine the sample. As such, 19 male and 19 female survivors were randomly sampled in each district, allowing the research team to determine a ‘pass/fail’ status for each district against benchmarks of interest that were nationally representative by gender. To account for districts with a disproportionately higher number of survivors, the research team decided to oversample survivors in the three districts with the highest numbers of EVD survivors.

#### Participatory approach

The research team had to be proactive and flexible in designing and implementing study protocols to overcome the intricate research challenges posed by the post-Ebola context of Sierra Leone. From its conception, the study aimed to use a participatory approach in order to understand the full picture of the post-Ebola context from all levels of stakeholders. However, utilizing a true participatory approach would involve the co-creation of research strategies and data collection tools with survivors themselves. This would have deeply biased the focus of the study and the data collected. To overcome this, the team liaised closely with SLAES and IPs who had direct access to survivors. They could help coordinate study activities to ensure access to the right mix of respondents, while still ensuring that they had no influence and/or participation in the selection of the study’s random sample of survivors or with conducting interviews, therefore limiting bias in the methodology or subsequent results.

#### Mixed methodology

Given the complex nature of Sierra Leone’s post-Ebola context, the research team purposely planned for a mixed-methods study that would include a large qualitative component, aimed to validate and complement the quantitative survey by diving deeper into important nuances that exist in multifaceted contexts. For example, the survey provided key information on referrals, access to health services, levels of disability, mental health status, etc. The qualitative data helped explain the reasons for these findings by probing into the views of program implementers and the district health teams. There are few studies to date with data on EVD survivors, making the need for such contextual information all the more important.

#### Integrate capacity building

Further, the ability to collect high-quality qualitative data is a specialized skill often difficult to find. This is even more the case in conflict and humanitarian settings. Capacity considerations not only drove the selection of interviewers to join the research team, but also greatly influenced the research training planning, which included extensive training on qualitative research methods; interviewing skills and administration of specialized mental health tools. Ensuring time for capacity building among the interviewers resulted in their improved ability to elicit nuanced information as well as effectively follow lines of inquiry. This effort, coupled with survey data, and the CPES PIU’s involvement and contextual insights helped build a fuller picture of survivors’ post-Ebola experiences and health needs while also creating a team of data collectors with improved data collection and research skills.

#### Contextualized assessment tools

The research team also took great care in administering the mental health and disability assessment tools. In order to minimize misinterpretation of the components of these tools by both the data collectors and respondents, the research team decided to use the short versions of the tools, which have also been validated globally. The data collection teams received extended training, including role-play, to ensure that they understood the purpose of the tools and could administer them accurately and consistently in each of the languages for data collection. Taking into account the nuances of the crisis context when adapting these tools, while also ensuring their sensitivity to the study population’s concerns was very important for accurate reporting and recording of findings.

## Conclusions

Conducting research in the post-Ebola setting led to a number of lessons learned. Most notably, the research team found that flexibility was paramount to conduct high-quality research that yielded representative and useful results. This included flexibility in the overall design of the research to follow a mixed-methods approach. Methods and protocols were also adapted to ensure that the research team was able to capture the appropriate sample of respondents using existing structures and/or networks. EVD survivors form a population that face stigma and have mental health and other long-term health needs that are only now being understood. Finding the appropriate yet flexible research methodology is key to accurately capture the sensitivities of this population.

Similarly, this research highlighted the importance of involving key stakeholders at multiple levels of the system. This involvement generated buy-in for this research on a study population covering some sensitive topics as well as enabled the appropriate adaptation and contextualization of the research tools and processes to the local situation. While these inputs are vital to collecting high-quality data, balancing the engagement of stakeholders in the process with the need to ensure independence and impartiality within the research design and subsequent data produced is an important and often difficult process that should be considered by research teams in the planning stages of a study.

Research in a post-crisis setting needs to be swift and responsive to allow for use of results to benefit or impact the affected populations. In these settings, research findings may go beyond the scope of the project or study focus, but should be considered rather than discarded with the recognition that people’s priorities are likely to be different from those of the researchers. In post-crisis settings, the most immediate needs are typically addressed. However, this is an opportunity for research to bring underlying issues to the surface, translating into more responsive solutions to impact target populations. Respondents need to be made aware of the research findings and its role in improving programs to address the study population’s needs, while also cautioning that not all of their issues would be resolved.

Findings from post-crisis settings can have a major impact on a country’s ability to address and resolve the existing crisis while also planning for future crises. However, sharing research findings beyond the research or project team often does not take place unless it is specifically mandated. The Sierra Leone project team prioritized continuously sharing results with the government, donors, and representatives at regional and national levels in order to better inform implementation priorities, demonstrating the importance of creating opportunities for sharing findings as they are obtained with all relevant levels of stakeholders.

## Data Availability

Anonymized survey data is available upon reasonable request by contacting the corresponding author, Dr. Soumya Alva: soumya_alva@jsi.com

## Abbreviations

CPES: Comprehensive Program for Ebola Survivors
EVD: Ebola Virus Disease
GAD: Generalized Anxiety Disorder
IP: Implementing Partner
LQAS: Lot Quality Assurance Sampling
MOHS: Ministry of Health and Sanitation
PHQ: Patient Health Questionnaire
PHU: Peripheral Health Unit
PIU: Program Implementation Unit
SLAES: Sierra Leone Association of Ebola Survivors
WHODAS-Short: World Health Organization Disability Assessment Schedule-short version

## Footnotes

1 During this period, Sierra Leone was comprised of 14 districts. However, two districts, Bonthe and Pujehun, with no or very few survivors did not receive significant support from CPES.
